# Synthesis of ∆^3^-2-Hydroxybakuchiol Analogues and Their Growth Inhibitory Activity against Rat UMR106 Cells

**DOI:** 10.3390/molecules19022213

**Published:** 2014-02-20

**Authors:** Qun Zhao, Qianqian Xu, Guangsheng Shan, Chao Dong, Hong Zhang, Xinsheng Lei

**Affiliations:** 1 School of Pharmacy, Fudan University, Shanghai 201203, China; 2 Department of Pharmaceutical Botany, School of Pharmacy, Second Military Medical University, Shanghai 200433, China; 3 Key Laboratory of Synthetic Chemistry of Natural Substances, Shanghai Institute of Organic Chemistry, Chinese Academy of Sciences, Shanghai 200032, China

**Keywords:** bakuchiol, natural product analogues, cytotoxic activity

## Abstract

A series of ∆^3^-2-hydroxybakuchiol analogues have been synthesized and tested for their growth inhibitory activity against rat UMR106 cells by using the MTT method. Some of them exhibit enhanced activities compared with the natural product, and the preliminary SAR profile shows that the chain tail on the natural product could be subtly modified to enhance the activity and the aromatic moiety or the terminal olefin on the main chain can also be modified without any evident loss of activity. The stereo-configuration of the quaternary chiral center has an important influence on the activity.

## 1. Introduction

**Figure 1:**
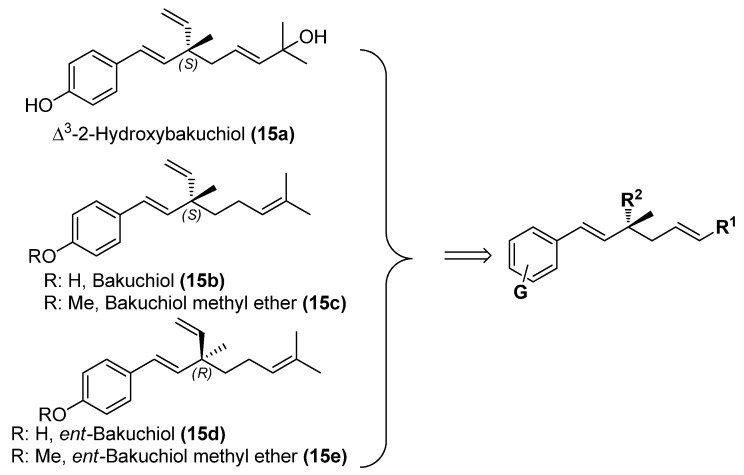
The structures of bakuchiol (**15b**) and ∆^3^-2-hydroxybakuchiol (**15a**) and the proposed modifications.

Natural products from plants are an important source of potential therapeutic agents for human health. The medicinal plant *Psoralea coryliforlia* L., a member of the Leguminosae family, has been used for a long time as a Traditional Chinese Medicine for the treatment of premature ejaculation, knee pain, pollakiuria, callus, psoriasis, vitiligo and psoriasis [1]. The seed extract has been suggested as a useful remedy for bone fractures, osteomalacia and osteoporosis [2]. A number of monoterpene phenols occurring in the plant have been isolated and demonstrated to possess interesting biological activities [3,4,5,6,7], and among them, bakuchiol (**15b**, Figure 1), one of the major components in the plant seed, has attracted great attention due to its diverse activities, such as antibacterial, antihelminthic, antioxidant, and especially antitumor properties [8,9,10,11,12,13,14,15,16,17,18,19,20,21,22,23]. In contrast to that, ∆^3^-2-hydroxybakuchiol (**15a**), a congener of bakuchiol, has not attracted much interest among medicinal chemists, probably due to its scarcity and lability [24,25]. To the best of our knowledge, only Guo and co-workers have recently investigated its inhibitory effects against monoamine transporters, suggesting that it might be a potential psychopharmacologic agent for the treatment of psychogenic disorders [26].

Given the fact that the analog bakuchiol shows various biological activities, we have put ∆^3^-2-hydroxybakuchiol into our natural product-based drug discovery program [27,28,29,30]. Recently, a facile asymmetric synthesis of ∆^3^-2-hydroxybakuchiol was established and the compound was tested for antiosteoporosis effects [31,32]. Unexpectedly, this natural product did not show the desired activity, but it exhibited growth inhibitory activity against osteosarcoma cells (rat UMR106 cell) with an IC_50_ value of 69 µM, suggesting that it was worthy of further investigation [33].

Herein the synthesis of ∆^3^-2-hydroxybakuchiol analogues was described and their growth inhibitory activity against rat UMR106 cells was demonstrated. The preliminary structure-activity relationship knowledge of this natural product has been obtained by modifying the substituent (G) on the aromatic ring and the R^1^ group on the chain, while keeping the main chain unchanged. In addition, we would also replace the terminal olefin with an ethyl moiety (R^2^) in order to probe the effect of this moiety on the activity (Figure 1).

## 2. Results and Discussion

### 2.1. Chemistry

**Scheme 1:**
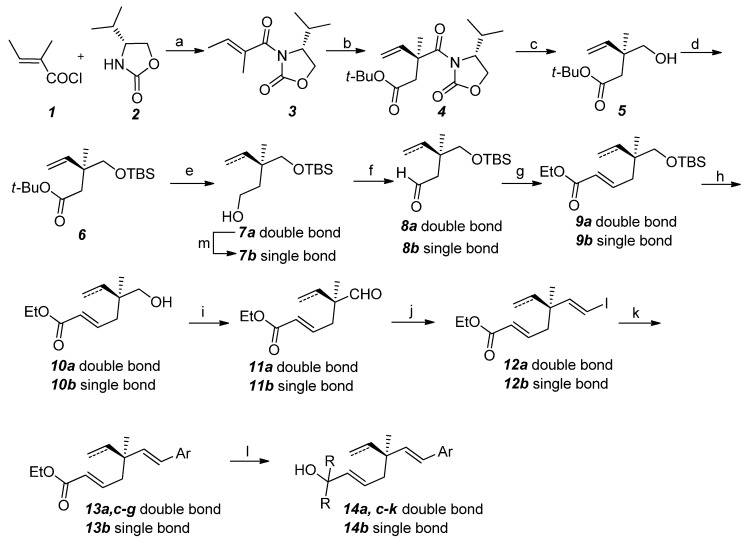
Synthetic approach to the analogues of ∆^3^-2-hydroxybakuchiol.

Our synthetic approach to the analogues of ∆^3^-2-hydroxybakuchiol is depicted in Scheme 1. Starting from (*E*)-2-methylbut-2-enoyl chloride and (*R*)-4-isopropyloxazolidin-2-one, the α,β-unsaturated imide **3** bearing an Evans’ auxiliary was prepared in excellent yield. Then, the a-alkylation of **3** with *tert*-butyl iodoacetate afforded the fragment **4** in a moderate yield with an excellent diastereoselectivity (dr > 20: 1). After selective reduction of the imide group, the key chiral intermediate **5** was obtained in an acceptable yield. To avoid the formation of the volatile lactone from **5**, the free hydroxyl group in **5** was protected with a TBS group to afford compound **6**. Upon reduction of the ester group and subsequent oxidation, compound **8a** was prepared in 94% yield (over two steps).

*Reagents and conditions*: (a) *n*-BuLi, THF, 96%; (b) NaHMDS, −78 ^○^C, 1.5 h; ICH_2_CO_2_Bu^t^, −50 ^○^C, 6 h, 68%; (c) LiBH_4_, THF, 0 ^○^C, 3 h, 70%; (d) TBSCl, imidazole, CH_2_Cl_2_, rt, 3 h, 84%; (e) DIBALH, CH_2_Cl_2_, −78 ^○^C, 2 h; (f) IBX, DMSO, rt, 5 h, for **8a**: 94%; for **8b**: 100%; two steps; (g) Ph_3_P=CHCO_2_Et, toluene, reflux, 2 h, for **9a**: 92%; for **9b**: 81%; (h) HCl, EtOH, 0 ^○^C, 7 h, for **10a**: 97%; for **10b**: 86%; (i) IBX, DMSO, rt, 5 h, for **11a**: 95%; for **11b**: 97%; (j) CHI_3_, CrCl_2_, THF, 0 ^○^C, 3 h, for **12a**: 86%; for **12b**: 80%; (k) ArBr, *n*-BuLi/ZnCl_2_; Pd(OAc)_2_/PPh_3_, THF, 60%–98%; (l) RLi, THF, rt, 5 h, up to 34%–100%; for **14b**, **14h**–**j**, additional step required for deprotection of TBDPS group: Bu_4_NF, THF, quantitative yield; (m) H_2_, Pd/C (10%), rt, overnight, 100%.

Compound **8a** successfully underwent a Wittig reaction to afford the α,β-unsaturated ester **9a**. Next, the TBS group was removed and oxidized to afford the corresponding aldehyde **11a** in 92% yield (over two steps). Through a Takai-Utimoto reaction with CrCl_2_ and CHI_3_, the *trans*-iodo-olefin **12a** was obtained in up to 86% yield. The iodo-olefin was coupled with different substituted aryl zinc species via the Negishi reaction to give the desired cross-coupling product **13a**,**c**–**f**, respectively. After selective 1,2-addition reactions of the esters **13a**, **c**–**g** with alkyllithiums, the corresponding products **14a**, **c**–**j** were obtained in acceptable (up to 73%) yields.

In order to assess the effect of the terminal olefin moiety in ∆^3^-2-hydroxybakuchiol on the biological activity, compound **14b** was prepared. Hydrogenation of **7a** provided saturated alcohol **7b** in a quantitative yield which was successfully transformed into **14b** by a method similar to that shown in Scheme 1. As positive control, bakuchiol (**15b**) and its methyl ether (**15c**), together with their corresponding enantiomers **15d** and **15e**, were also prepared according to our recently reported method [31].

### 2.2. Activity against Rat UMR106 Cell

With the various analogues of ∆^3^-2-hydroxybakuchiol in hand, their cytotoxic activity against rat UMR106 osteosarcoma cells was tested by the MTT assay after two days of treatment [34] and the results are shown in Table 1.

Among those compounds, ∆^3^-2-hydroxybakuchiol (**15a**) displayed the expected [33] cytotoxic activity with an IC_50_ value of 69 µM (Entry 21, Table 1). When the chain tail was modified with an α,β-unsaturated ester (compound **13a**), the activity increased (IC_50_: 27 µM, Entry 1) compared with **14a** (IC_50_: 66 µM, Entry 7). When the methoxy group was replaced with other substituent (compounds **13c**–**g**), the corresponding activity was significantly reduced (IC_50_: > 263 µM, Entries 2–6), suggesting that the substituent on the aromatic ring had an important influence on the activity in the case of α,β-unsaturated ester analogues.

When the modifications were performed only on the aromatic moiety of ∆^3^-2-hydroxybakuchiol, the substituent effect seemed to have somewhat of an effect on the activity (compounds **14a**, **c**, **d**, **f**). For example, the activity was still retained in the case of a *para*-methoxy (**14a**; Entry 7), but other groups such as H and *para*-methyl led to slightly reduced activities (**14c**, **14d**; Entries 8 and 9). Notably, a strong electron-withdrawing group gave a slightly increased activity (**14f**, Entry 11). When a larger bulky group was used (compounds **14e**,**g**; Entries 10 and 12), the activity was just slightly decreased. Surprisingly, if the phenolic hydroxyl was moved to the *meta*-position (**14h**), its IC_50_ value was still up to 123 µM, with about one-fold reduced activity in contrast to ∆^3^-2-hydroxybakuchiol (Entry 13). Modification on the R^1^ group (**14i**,**j**) in the tertiary alcohol moiety suggested that this domain could accommodate a bulky hydrophobic space, and **14j** gave the best result (IC_50_: 17 µM; Entries 14 and 15). In addition, the terminal double bond could be saturated without influence on the activity, implied by the almost identical activities of **14b** (IC_50_: 71 µM; Entry 16) and ∆^3^-2-hydroxybakuchiol. 

On the other hand, bakuchiol (**15b**), with a slight structural difference on the chain tail of ∆^3^-2-hydroxybakuchiol, showed almost the same potency (IC_50_: 62 µM, Entry 18). Interestingly, *ent*-bakuchiol (**15d**) had a one-fold enhanced activity (IC_50_: 33 µM, Entry 20). However, in comparison with (*S*- or *R*)bakuchiol methyl ether showed different influences on the activity, namely, (*S*)-bakuchiol methyl ether (**15c)** maintained the activity (IC_50_: 60 µM, Entry 17) but (*R*)-bakuchiol methyl ether (**15e**) gave less activity (IC_50_: 161 µM, Entry 19), indicating that the stereochemistry played an important role to the activity.

**Table 1:** Cytotoxic activities of ∆^3^-2-hydroxybakuchiol and its analogues against rat UMR106 osteosarcoma cell.

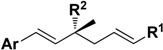						
Entry	Comp.	Ar		R^1^	R^2^	IC_50 _(µM)
**1**	**13a**	4-MeO-C_6_H_4_		CO_2_Et	CH=CH_2_	27
**2**	**13c**	C_6_H_5_		CO_2_Et	CH=CH_2_	330
**3**	**13d**	4-Me-C_6_H_4_		CO_2_Et	CH=CH_2_	263
**4**	**13e**	2-C_10_H_7_		CO_2_Et	CH=CH_2_	>500
**5**	**13f**	4-CF_3_-C_6_H_4_		CO_2_Et	CH=CH_2_	242
**6**	**13g**	3,4-OCH_2_O-C_6_H_4_		CO_2_Et	CH=CH_2_	511
**7**	**14a**	4-MeO-C_6_H_4_		Me_2_CH(OH)	CH=CH_2_	66
**8**	**14c**	C_6_H_5_		Me_2_CH(OH)	CH=CH_2_	107
**9**	**14d**	4-Me-C_6_H_4_		Me_2_CH(OH)	CH=CH_2_	87
**10**	**14e**	2-C_10_H_7_		Me_2_CH(OH)	CH=CH_2_	128
**11**	**14f**	4-CF_3_-C_6_H_4_		Me_2_CH(OH)	CH=CH_2_	46
**12**	**14g**	3,4-OCH_2_O-C_6_H_4_		Me_2_CH(OH)	CH=CH_2_	115
**13**	**14h**	3-HO-C_6_H_4_		Me_2_CH(OH)	CH=CH_2_	123
**14**	**14i**	4-HO-C_6_H_4_		Et_2_CH(OH)	CH=CH_2_	57
**15**	**14j**	4-HO-C_6_H_4_		*n*-Bu_2_CH(OH)	CH=CH_2_	17
**16**	**14b**	4-HO-C_6_H_4_		Me_2_CH(OH)	CH_2_CH_3_	71
**17**	bakuchiol methyl ether (**15c**)		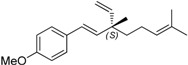			60
**18**	bakuchiol (**15b**)		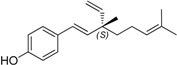			62
**19**	*ent*-bakuchiol methyl ether (**15e**)		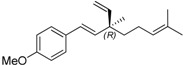			161
**20**	*ent*-bakuchiol (**15d**)		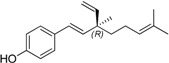			33
**21**	∆^3^-2-hydroxybakuchiol (**15a**)		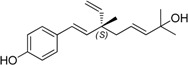			69

## 3. Experimental

### 3.1. General Information

Solvents were distilled from the appropriate drying agents before use. All the reagents were purchased from Acros (Shanghai, China), Alfa Aesar (Shanghai, China), and National Chemical Reagents Group Co. Ltd (Shanghai, China). Unless otherwise stated, all the reactions were performed under argon. Column chromatography: commercial silica gel (Qingdao Haiyang Chemical Group Co.; 300–400 mesh, Qingdao, China). Spots on the TLC plates (GF 254, Yantai Jiangyou Silica R&D Co. Ltd., Yantai, China) were detected under UV light or with iodine, KMnO_4_, and H_3_PO_4_·12MoO_3_·xH_2_O. ^1^H and ^13^C-NMR spectra (400 MHz for ^1^H, and 100 MHz for ^13^C): Varian Mercury-Plus (Palo Alto, CA, USA) spectrometer; chemical shifts δ in parts per million, with residual CHCl_3_ [d (H) 7.26; d (C) 77.0] as internal standard; *J* in Hertz. ESI-MS and HR-APCI/ESI-MS: Finnigan Mat-95 mass spectrometer (Waltham, MA, USA); in *m/z*. Compounds **3**–**7a**, **8**–**14a**, ∆^3^-2-hydroxybakuchiol, (*S*)-bakuchiol and (*R*)-bakuchiol as well as their corresponding methyl ethers (**15a**–**e**) were prepared by our previously reported method [32].

### 3.2. Synthetic Procedures for the New Compounds

*(R)-3-(((tert-Butyldimethylsilyl)oxy)methyl)-3-methylpentan-1-ol* (**7b**). A mixture of (S)-3-(((*tert*-butyldimethylsilyl)oxy)methyl)-3-methylpent-4-en-1-ol (1.0 g, 4.09 mmol) and Pd/C (100 mg, 10% *w/w*) in methanol (20 mL) was purged with H_2_ gas and was stirred overnight at room temperature under H_2_. The reaction mixture was directly passed through a pad of silica gel with ethyl acetate and the solvent was removed under vacuum. The residue was purified by column chromatography (petroleum ether/ethyl acetate 10:1) to afford **7b** (1.01 g, 100%) as a colorless oil. *R*_f_ = 0.26 (petroleum ether/ethyl acetate 10:1), 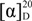 +4.8 (c 0.65, CHCl_3_). ^1^H-NMR (CDCl_3_) δ 3.64 (t, *J* = 5.6 Hz, 2H), 3.41 (brs, 1H), 3.39 (d, *J* = 10.0 Hz, 1H), 3.34 (d, *J* = 10.0 Hz, 1H), 1.64–1.48 (m, 2H), 1.39–1.20 (m, 2H), 0.91 (s, 9H), 0.85–0.78 (m, 6H), 0.08 (s, 6H). ^13^C-NMR (CDCl_3_) δ 70.6, 58.8, 41.2, 37.4, 29.6, 25.8, 21.7, 18.2, 7.7, −5.6. ESI-MS: 247.1 [M+H], HRMS (ESI): Calcd. for C_13_H_30_O_2_Si [M+H]: 247.2088, found: 247.2091.

*(R)-3-(((tert-Butyldimethylsilyl)oxy)methyl)-3-methylpentanal* (**8b**). IBX (1.71 g, 4.9 mmol) was added to a solution of **7b** (1.01 g, 4.09 mmol) in DMSO (20 mL). The mixture was stirred overnight at room temperature. Then the mixture was diluted with ethyl acetate (200 mL) and washed successively with saturated NaHCO_3_ aq. solution (30 mL × 2), water (30 mL), brine (30 mL), dried over anhydrous Na_2_SO_4_, filtered and concentrated under reduced pressure. The residue was purified by column chromatography (petroleum ether/ethyl acetate 20:1) to afford **8b** (1.00 g, 99%) as a colorless oil. *R*_f_ = 0.74 (petroleum ether/ethyl acetate = 10:1), 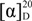 −2.6 (c 0.50, CHCl_3_). ^1^H-NMR (CDCl_3_) δ 9.84 (t, *J* = 3.1 Hz, 1H), 3.42 (d, *J* = 9.7 Hz, 1H), 3.35 (d, *J* = 9.7 Hz, 1H), 2.32–2.21 (m, 2H), 1.46–1.37 (m, 2H), 0.96 (s, 3H), 0.92–0.83 (m, 12H), 0.03 (s, 6H). ^13^C-NMR (CDCl_3_) δ 203.8, 69.6, 51.0, 42.2, 37.8, 29.4, 25.8, 21.3, 7.9, −5.6. ESI-MS: 283.7 [M+K]. 

*(R,E)-Ethyl 5-(((tert-butyldimethylsilyl)oxy)methyl)-5-methylhept-2-enoate* (**9b**). To a solution of **8b** (0.978 g, 4.0 mmol) in toluene (10 mL) was added a solution of ethyl-2-(triphenyl-phosphoranylidene)acetate (1.71 g, 4.9 mmol) in toluene (10 mL). The mixture was stirred for 2 h under argon at reflux and then cooled to rt. The reaction mixture was concentrated under reduced pressure, diluted with ether (50 mL) and filtered. The filter cake was washed successively with ether (10 mL) and petroleum ether (10 mL). The combined filtrate was concentrated under reduced pressure. The residue was purified by column chromatography (petroleum ether/ethyl acetate 80:1) to afford **9b** (0.953 g, 81%) as a colorless oil. *R*_f_ = 0.60 (petroleum ether/ethyl acetate = 20:1), 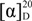 −11.5 (c 0.60, CHCl_3_). ^1^H-NMR (CDCl_3_) δ 6.97 (dt, *J* = 15.7, 7.9 Hz, 1H), 5.81 (d, *J* = 15.7 Hz, 1H), 4.18 (q, *J* = 7.1 Hz, 2H), 3.28 (d, *J* = 9.7 Hz, 1H), 3.23 (d, *J* = 9.7 Hz, 1H), 2.14 (d, *J* = 7.9 Hz, 2H), 1.28 (t, *J* = 7.1 Hz, 3H), 0.89 (s, 9H), 0.85–0.76 (m, 6H), 0.02 (s, 6H). ^13^C-NMR (CDCl_3_) δ 166.6, 146.8, 123.2, 68.6, 60.1, 39.4, 38.8, 29.0, 3.82, 21.1, 18.2, 14.3, 7.8, −5.6. ESI-MS: 315.2 [M+H], HRMS(ESI): Calcd. for C_17_H_35_O_3_Si [M+H]: 315.2350, found: 315.2356.

*(R,E)-Ethyl-5-(hydroxymethyl)-5-methylhept-2-enoate* (**10b**). To a solution of **9b** (950 mg, 3.02 mmol) in methanol (10 mL) at 0 ^○^C was added concentrated hydrochloric acid (0.75 mL). The mixture was stirred for 7 h at rt. The reaction was quenched with saturated NaHCO_3_ (10 mL). The mixture was concentrated under reduced pressure. The residue was extracted with ethyl acetate (15 mL × 3). The organic phase was combined, washed with brine (15 mL), dried over anhydrous Na_2_SO_4_, filtered, and the filtrate was concentrated under reduced pressure. The residue was purified by column chromatography (petroleum ether/ethyl acetate 20:1) to afford **10b** (517 mg, 86%) as a colorless oil. *R*_f_ = 0.81 (petroleum ether/ethyl acetate = 5:1), 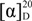 −14.7 (c 0.60, CHCl_3_). ^1^H-NMR (CDCl_3_) δ 7.10–6.88 (m, 1H), 5.86 (ddd, *J* = 15.5, 3.0, 1.7 Hz, 1H), 4.19 (q, *J* = 7.1 Hz, 2H), 3.40 (d, *J* = 10.8 Hz, 1H), 3.36 (d, *J* = 10.8 Hz, 1H), 2.18 (dd, *J* = 7.9, 1.3 Hz, 2H), 1.37–1.25 (m, 5H), 0.91–0.81 (m, 6H). ^13^C-NMR (CDCl_3_) δ 166.4, 146.0, 123.6, 68.9, 60.2, 39.1, 38.6, 28.8, 21.1, 14.33, 7.8. ESI-MS: 201.1 [M+H], HRMS(ESI): Calcd. for C_11_H_21_O_3_ [M+H]: 201.1485, found: 201.1489.

*(R,E)-Ethyl 5-formyl-5-methylhept-2-enoate* (**11b**). Compound **10b** (447 mg, 2.23 mmol) and IBX (938 mg, 3.4 mmol) in a 100 mL flask was dissolved with DMSO (20 mL). The mixture was stirred overnight at rt. Then the mixture was diluted with ethyl acetate (300 mL), washed successively with saturated NaHCO_3_ aq. solution (40 mL × 2), water (40 mL), brine (40 mL), dried over anhydrous Na_2_SO_4_, filtered and concentrated under reduced pressure. The residue was purified by column chromatography (petroleum ether/ethyl acetate 20:1) to afford **11b** (430 mg, 97%) as a colorless oil. *R*_f _= 0.74 (petroleum ether/ethyl acetate = 5:1), 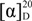 −21.6 (c 0.45, CHCl_3_) ^1^H-NMR (CDCl_3_) δ 7.04–6.89 (m, 1H), 5.86 (ddd, *J* = 15.5, 3.0, 1.7 Hz, 1H), 4.19 (q, *J* = 7.1 Hz, 2H), 3.40 (d, *J* = 10.8 Hz, 1H), 3.36 (d, *J* = 10.8 Hz, 1H), 2.18 (dd, *J* = 7.9, 1.3 Hz, 2H), 1.44–1.22 (m, 5H), 0.92–0.79 (m, 6H). ^13^C-NMR (CDCl_3_) δ 205.3, 166.0, 143.6, 124.6, 60.4, 49.3, 36.7, 27.9, 18.6, 14.2, 8.3. ESI-MS: 237.1, [M+K] HRMS(ESI): Calcd. for C_11_H_19_O_3_ [M+H]: 199.1329, found: 199.1331.

*(R,2E,6E)-Ethyl 5-ethyl-7-iodo-5-methylhepta-2,6-dienoate* (**12b**). To a suspension of anhydrous CrCl_2_ (1.85 g, 8.82 mmol) in THF (20 mL) at 0 ^○^C was added dropwise a solution of **11b** (388 mg, 2.0 mmol) and CHI_3_ (1.16 g, 2.9 mmol) in THF (10 mL). After 3 h, the mixture was quenched with 10% Na_2_S_2_O_3_ aq. solution (20 mL). The mixture was extracted with ethyl acetate (20 mL × 3). The combined organic phase was washed with brine (20 mL), dried over anhydrous Na_2_SO_4_, filtered, and the filtrate was concentrated under reduced pressure. The residue was purified by column chromatography (petroleum ether/ethyl acetate 50:1) to afford **12b** (504 mg, 80%) as a colorless oil. *R*_f_ = 0.61 (petroleum ether/ethyl acetate = 10:1), 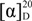 −39.2 (c 0.65, CHCl_3_) ^1^H-NMR (CDCl_3_) δ 6.96–6.75 (m, 1H), 6.44 (d, *J* = 14.7 Hz, 1H), 5.97 (d, *J* = 14.7 Hz, 1H), 5.82 (d, *J* = 15.5 Hz, 1H), 4.19 (q, *J* = 7.1 Hz, 2H), 2.26–2.13 (m, 2H), 1.43–1.24 (m, 5H), 0.98 (s, 3H), 0.82 (t, *J* = 7.1 Hz, 3H). ^13^C-NMR (CDCl_3_) δ 166.2, 153.2, 144.8, 124.0, 74.3, 60.2, 44.3, 42.6, 32.6, 22.1, 14.2, 8.4. ESI-MS: 323.0 [M+H], HRMS(ESI): Calcd. for C_12_H_20_IO_2_ [M+H]: 323.0502, found: 323.0511.

General procedure for the synthesis of compounds **13b**–**g**, exemplified by *(R,2E,6E)-ethyl 7-(4-((tert-butyldiphenylsilyl)oxy)phenyl)-5-ethyl-5-methylhepta-2,6-dienoate* (**13b)**. A dried flask was charged with ZnCl_2_ (300 mg, 2.2 mmol), and heated by a hot gun under vacuum until the ZnCl_2_ was melted. Then the flask was filled with Ar gas and cooled to rt. Anhydrous THF (5 mL) was added into the flask to dissolve the ZnCl_2_. Another dried flask was charged with 1-bromo-4-(*t*-BuPh_2_SiO)-C_6_H_4_Br (823 mg, 2.0 mmol) and THF (5 mL) under argon, and then cooled to −78 ^○^C. *n*-BuLi (0.8 mL, 2.0 mmol) was added dropwise to the bromoarene solution over 30 min, and stirring was continued for 30 min. Then the organolithium solution was added dropwise to the ZnCl_2_ solution at −78 ^○^C over 15 min, and the resulting solution was stirred at rt for 1 h. Meanwhile, a mixture of [Pd(OAc)_2_] (22 mg, 0.1 mmol), PPh_3_ (26 mg, 0.1 mmol) and THF (2 mL) was stirred at rt for about 30 min until the brown solution was formed, and this solution was added to the organozinc solution, followed by a solution of the **12b** (322 mg, 1.0 mmol) in THF (2 mL). The mixed solution was stirred overnight at rt. The reaction solution was quenched with saturated NH_4_Cl aq. solution (15 mL), and extracted with ethyl acetate (15 mL × 3). The extract was washed with brine (15 mL), dried over anhydrous Na_2_SO_4_, and filtered. The filtrate was concentrated under reduced pressure. The residue was purified by column chromatography (petroleum ether/ethyl acetate 80:1) to afford **13b** (463 mg, 88%) as a colorless oil. *R*_f_ = 0.43 (petroleum ether/ethyl acetate = 20:1), 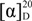 −40.4 (c 0.50, CHCl_3_). ^1^H-NMR (CDCl_3_) δ 7.77–7.66 (m, 4H), 7.46–7.31 (m, 6H), 7.10 (d, *J* = 8.4 Hz, 2H), 6.91 (dt, *J* = 15.4, 7.6 Hz, 1H), 6.70 (d, *J* = 8.4 Hz, 2H), 6.16 (d, *J* = 16.3 Hz, 1H), 5.90 (d, *J* = 16.3 Hz, 1H), 5.81 (d, *J* = 15.5 Hz, 1H), 4.16 (q, *J* = 7.1 Hz, 2H), 2.31–2.18 (m, 2H), 1.49–1.33 (m, 2H), 1.26 (t, *J* = 7.1 Hz, 3H), 1.09 (s, 9H), 1.03 (s, 3H), 0.80 (t, *J* = 7.4 Hz, 3H). ^13^C-NMR (CDCl_3_) δ 166.4, 154.8, 146.2, 135.7, 135.5, 132.9, 130.6, 129.9, 127.8, 127.2, 126.9, 123.4, 119.6, 60.1, 43.7, 39.6, 33.5, 26.5, 19.4, 14.3, 8.5. ESI-MS: 527.2 [M+H].

*(S,2E,6E)-Ethyl 5-methyl-7-phenyl-5-vinylhepta-2,6-dienoate* (**13c**). Yield: 98%, *R*_f_ = 0.43 (petroleum ether/ethyl acetate = 20:1), 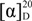 −3.1 (c 0.55, CHCl_3_). ^1^H-NMR (CDCl_3_) δ 7.39–7.17 (m, 5H), 6.93 (dt, *J* = 15.4, 7.6 Hz, 1H), 6.36 (d, *J* = 16.2 Hz, 1H), 6.20 (d, *J* = 16.2 Hz, 1H), 5.93–5.82 (m, 2H), 5.11 (d, *J* = 10.7 Hz, 1H), 5.06 (d, *J* = 17.5 Hz, 1H), 4.19 (q, *J* = 7.1 Hz, 2H), 2.41 (d, *J* = 7.6 Hz, 2H), 1.28 (t, *J* = 7.1 Hz, 3H), 1.23 (s, 3H). ^13^C-NMR (CDCl_3_) δ 166.3, 145.3, 144.4, 137.3, 136.4, 128.5, 128.1, 127.2, 126.2, 123.9, 113.1, 60.2, 43.8, 42.62, 23.7, 14.2. ESI-MS: 271.2 [M+H], 293.1 [M+Na], HRMS (ESI): Calcd. for C_18_H_22_O_2_Na [M+H]: 293.1512, found: 293.1518.

*(S,2E,6E)-Ethyl-5-methyl-7-(p-tolyl)-5-vinylhepta-2,6-dienoate* (**13d**) Yield: 82%, *R*_f_ = 0.41 (petroleum ether/ethyl acetate =20:1), 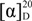 +3.3 (c 0.55, CHCl_3_). ^1^H-NMR (CDCl_3_) δ 7.25 (d, *J* = 7.7 Hz, 2H), 7.11 (d, *J* = 7.7 Hz, 2H), 6.92 (dt, *J* = 15.4, 7.6 Hz, 1H), 6.32 (d, *J* = 16.2 Hz, 1H), 6.14 (d, *J* = 16.2 Hz, 1H), 5.94–5.77 (m, 2H), 5.12–5.01 (m, 2H), 4.17 (q, 7.2 Hz, 2H), 2.40 (d, *J* = 7.4 Hz, 2H), 2.33 (s, 3H), 1.28 (t, *J* = 7.2 Hz, 3H), 1.21 (s, 3H). ^13^C-NMR (CDCl_3_) δ 166.3, 145.5, 144.6, 137.0, 135.4, 134.5, 129.2, 127.9, 126.1, 123.8, 113.0, 60.2, 43.8, 42.6, 23.7, 21.1, 14.2. ESI-MS: 307.1 [M+Na], HRMS(ESI): Calcd. for C_19_H_25_O_2_ [M+H]: 285.1849, found: 285.1855.

*(S,2E,6E)-ethyl 5-methyl-7-(naphthalen-2-yl)-5-vinylhepta-2,6-dienoate* (**13e**) Yield: 60%, *R*_f_ = 0.34 (petroleum ether/ethyl acetate =20:1), 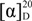 −4.4 (c 0.50, CHCl_3_). ^1^H-NMR (CDCl_3_) δ 7.84–7.74 (m, 3H), 7.71 (s, 1H), 7.59 (d, *J* = 8.6 Hz, 1H), 7.50–7.37 (m, 2H), 6.96 (dt, *J* = 15.4, 7.5 Hz, 1H), 6.52 (d, *J* = 16.2 Hz, 1H), 6.33 (d, *J* = 16.2 Hz, 1H), 6.00–5.82 (m, 2H), 5.17–5.05 (m, 2H), 4.18 (q, 7.1 Hz, 2H), 2.46 (d, *J* = 7.5 Hz, 2H), 1.30–1.23 (m, 6H). ESI-MS: 343.3 [M+Na], HRMS (ESI): Calcd. for C_22_H_25_O_2_ [M+H]: 321.1849, found: 321.1857.

*(S,2E,6E)-Ethyl-5-methyl-7-(4-(trifluoromethyl)phenyl)-5-vinylhepta-2,6-dienoate* (**13f**). Yield: 90%, *R*_f_ = 0.41 (petroleum ether/ethyl acetate = 20:1), 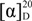 +9.8 (c 0.50, CHCl_3_). ^1^H-NMR (CDCl_3_) δ 7.55 (d, *J* = 8.2 Hz, 2H), 7.45 (d, *J* = 8.2 Hz, 2H), 6.98–6.84 (m, 1H), 6.39 (d, *J* = 16.2 Hz, 1H), 6.29 (d, *J* = 16.2 Hz, 1H), 5.96–5.82 (m, 2H), 5.20–5.11 (m, 1H), 5.08 (dd, *J* = 17.4, 0.8 Hz, 1H), 4.18 (q, *J* = 7.1 Hz, 2H), 2.43 (dd, *J* = 7.6, 1.3 Hz, 2H), 1.28 (t, *J* = 7.1 Hz, 3H), 1.24 (s, 3H). ^13^C-NMR (CDCl_3_) δ 166.2 (C4), 144.9 (C7), 143.9 (C11), 140.8 (C15), 139.1 (C14), 128.9 (C18), 127.0 (C10), 126.3 (C16), 125.5 (C17), 124.1 (C5), 113.6 (13), 60.3 (C2), 43.6 (C8), 42.8 (C9), 23.5 (C12), 14.2 (C1).ESI-MS: 361.2 [M+Na], HRMS(ESI): Calcd. for C_19_H_22_F_3_O_2_ [M+H]: 339.1566, found: 339.1575.

*(S,2E,6E)-Ethyl-7-(benzo[d]* [1,3] *dioxol-5-yl)-5-methyl-5-vinylhepta-2,6-dienoate* (**13g**). Yield: 96%, *R*_f_ = 0.30 (petroleum ether/ethyl acetate = 20:1), 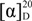 −15.0 (c 0.50, CHCl_3_). ^1^H-NMR (CDCl_3_) δ 6.97–6.86 (m, 2H, C(7)H, C(20)H), 6.81–6.71 (m, 2H, C(16)H, C(17)H), 6.26 (d, *J* = 16.2 Hz, 1H, C(14)H), 6.02 (d, *J* = 16.2 Hz, 1H, C(10)H), 5.94 (s, 2H, C(22)H), 5.92–5.81 (m, 2H, C(5)H, C(11)H), 5.10 (dd, *J* = 10.7, 0.7 Hz, 1H, C(13)H), 5.05 (d, *J* = 17.5 Hz, 1H, C(13)H), 4.18 (q, *J* = 7.1 Hz, 2H, C(2)H2), 2.42–2.36 (m, 2H, C(8)H), 1.28 (t, *J* = 7.1 Hz, 3H, C(1)H), 1.20 (s, 3H, C(12)H3). ^13^C-NMR (CDCl_3_) δ 166.3 (C(4)), 147.9 (C(19)), 146.9 (C(18)), 145.4 (C(7)), 144.5 (C(11)), 134.7 (C(14)), 131.8 (C(15)), 127.6 (C(10)), 123.8 (C(5)), 120.7, 113.0 (C(13)), 108.2, 105.5, 101.0 (C(16), C(17), C(20)), 60.2 (C(2)), 43.8 (C(8)), 42.5 (C(9)), 23.7 (C(12)), 14.2 (C(1)). ESI-MS: 315.2[M+H], 337.2 [M+Na], HRMS(ESI): Calcd. for C_19_H_23_O_4_ [M+H]: 315.1591, found: 315.1602.

General procedure for the synthesis for **14b**–**g**, exemplified by *4-((R,1E,5E)-3-ethyl-7-hydroxy-3,7-dimethylocta-1,5-dien-1-yl)phenol* (**14b**). To a stirred solution of **13b** (267 mg, 0.51 mmol,) in THF (5 mL) at 0 ^○^C was added MeLi (1.0 mL, 1.5 mmol). The mixture was stirred overnight at rt. The reaction was quenched with saturated NH_4_Cl (10 mL). The mixture was extracted with ethyl acetate (10 mL × 3). The combined organic phase was washed with brine (10 mL), dried over anhydrous Na_2_SO_4_, filtered, and the filtrate was concentrated under reduced pressure. The residue was purified by column chromatography (petroleum ether/ethyl acetate 5:1) to afford the silyl protected **14b** (124 mg, 48%) together with **14b** (47 mg, 34%) as a colorless oil. The protected compound could be deprotected quantitatively into **14b** by *n*-Bu_4_NF in THF at rt for 3–4 h.

Silyl protected **14b**: *R*_f_ = 0.45 (petroleum ether/ethyl acetate = 5:1), 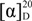 −65.5 (c 0.20, CHCl_3_). ^1^H-NMR (CDCl_3_) δ 7.76–7.67 (m, 4H), 7.46–7.31 (m, 6H), 7.09 (d, *J* = 8.6 Hz, 2H), 6.69 (d, *J* = 8.6 Hz, 2H), 6.12 (d, *J* = 16.3 Hz, 1H), 5.90 (d, *J* = 16.2 Hz, 1H), 5.64–5.50 (m, 2H), 2.12–1.98 (m, 2H), 1.40–1.32 (m, 2H), 1.27 (s, 6H), 1.09 (s, 9H), 0.98 (s, 3H), 0.79 (t, *J* = 7.4 Hz, 3H). ^13^C-NMR (CDCl_3_) δ 154.6, 140.6, 136.9, 135.5, 132.9, 130.9, 129.8, 127.7, 126.8, 126.5, 123.4, 119.6, 70.7, 43.6, 39.4, 33.3, 29.9, 26.5, 22.9, 19.4, 8.6. ESI-MS: 530.2 [M+NH_4_].

**14b**: *R*_f_ = 0.09 (petroleum ether/ethyl acetate = 5:1), 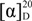 −36.3 (c 0.07, CHCl_3_). ^1^H-NMR (CDCl_3_) δ 7.23 (d, *J* = 8.6 Hz, 2H), 6.77 (d, *J* = 8.6 Hz, 2H), 6.18 (d, *J* = 16.3 Hz, 1H), 5.96 (d, *J* = 16.3 Hz, 1H), 5.68–5.49 (m, 2H), 2.17–2.03 (m, 2H), 1.46–1.34 (m, 2H), 1.30 (s, 6H), 1.02 (s, 3H), 0.82 (t, *J* = 7.5 Hz, 3H). ^13^C-NMR (CDCl_3_) δ 154.7, 140.5, 136.8, 130.8, 127.2, 126.4, 123.5, 115.3, 70.9, 43.6, 39.5, 33.3, 29.8, 22.8, 8.6. ESI-MS: 530.2 [M+NH_4_].

*(S,3E,7E)-2,6-Dimethyl-8-phenyl-6-vinylocta-3,7-dien-2-ol* (**14c**) Yield: 73%, *R*_f_ = 0.42 (petroleum ether/ethyl acetate = 5:1), 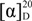 −9.5 (c 0.55, CHCl_3_). ^1^H-NMR (CDCl_3_) δ 7.36 (d, *J* = 8.0 Hz, 2H), 7.33–7.25 (m, 2H), 7.20 (t, *J* = 6.6 Hz, 1H), 6.32 (d, *J* = 16.2 Hz, 1H), 6.20 (d, *J* = 16.2 Hz, 1H), 5.89 (dd, *J* = 17.4, 10.7 Hz, 1H), 5.70–5.55 (m, 2H), 5.09–4.98 (m, 2H), 2.23 (d, *J* = 6.3 Hz, 2H), 1.38 (s, 1H), 1.30 (s, 6H), 1.18 (s, 3H). ^13^C-NMR (CDCl_3_) δ 145.3, 141.2, 137.6, 137.4, 128.5, 127.4, 127.0, 126.1, 122.9, 112.3, 70.7, 43.8, 42. 7, 29.9, 23.4. ESI-MS: 274.2 [M+NH_4_], HRMS (ESI): Calcd. for C_18_H_25_O [M+H]: 257.1900, found: 257.1899.

*(S,3E,7E)-2,6-Dimethyl-8-(p-tolyl)-6-vinylocta-3,7-dien-2-ol* (**14d**) Yield: 98%, *R*_f_ = 0.46 (petroleum ether/ethyl acetate = 5:1), 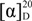 −9.1 (c 0.55, CHCl_3_). ^1^H-NMR (CDCl_3_) δ 7.25 (d, *J* = 7.3 Hz, 2H), 7.10 (d, *J* = 7.3 Hz, 2H), 6.28 (d, *J* = 16.2 Hz, 1H), 6.14 (d, *J* = 16.2 Hz, 1H), 5.88 (dd, *J* = 17.4, 10.7 Hz, 1H), 5.70–5.53 (m, 2H), 5.11–4.94 (m, 2H), 2.32 (s, 3H), 2.22 (d, *J* = 5.9 Hz, 2H), 1.42 (brs, 1H), 1.29 (s, 6H), 1.17 (s, 3H). ^13^C-NMR (CDCl_3_) δ 145.4, 141.1, 136.7, 136.4, 134.9, 129.2, 127.3, 126.0, 123.0, 112.2, 70.7, 43.8, 42.6, 29.8, 23.4, 21.1. ESI-MS: 293.2 [M+Na].

*(S,3E,7E)-2,6-Dimethyl-8-(naphthalen-2-yl)-6-vinylocta-3,7-dien-2-ol* (**14e**) Yield: 100%, *R*_f_ = 0.41 (petroleum ether/ethyl acetate = 5:1), 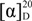 −13.7 (c 0.35, CHCl_3_). ^1^H-NMR (CDCl_3_) δ 7.83–7.74 (m, 3H), 7.70 (s, 1H), 7.59 (d, J = 8.6 Hz, 1H), 7.48–7.38 (m, 2H), 6.49 (d, *J* = 16.2 Hz, 1H), 6.34 (dd, *J* = 16.2, 2.0 Hz, 1H), 5.93 (ddd, *J* = 17.4, 10.7, 2.0 Hz, 1H), 5.72-5.57 (m, 2H), 5.14–5.01 (m, 2H), 2.26 (dd, *J* = 5.9, 1.7 Hz, 2H), 1.41 (s, 1H), 1.30 (s, 6H), 1.23 (s, 3H). ^13^C-NMR (CDCl_3_) δ 145.3, 141.2, 137.9, 135.1, 133.6, 132.7, 128.0, 127.8, 127.6, 126.1, 125.68, 125.5, 123.5, 122.9, 112.4, 70.8, 43.8, 42.8, 29.9, 23.4. ESI-MS: 293.2 [M+Na], HRMS (ESI): Calcd. for C_22_H_26_ONa [M+Na]: 329.1876, found: 329.1885.

*(S,3E,7E)-2,6-Dimethyl-8-(4-(trifluoromethyl)phenyl)-6-vinylocta-3,7-dien-2-ol* (**14f**) Yield: 96%, *R*_f_ = 0.42 (petroleum ether/ethyl acetate = 5:1), 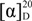 +2.0 (c 0.75, CHCl_3_). ^1^H-NMR (CDCl_3_) δ 7.54 (d, *J* = 8.2 Hz, 2H), 7.44 (d, *J* = 8.2 Hz, 2H), 6.35 (d, *J* = 16.3 Hz, 1H), 6.30 (d, *J* = 16.3 Hz, 1H), 5.89 (dd, *J* = 17.5, 10.7 Hz, 1H), 5.70–5.53 (m, 2H), 5.09 (dd, *J* = 10.7, 1.0 Hz, 1H), 5.03 (dd, *J* = 17.5, 1.0 Hz, 1H), 2.24 (d, *J* = 6.6 Hz, 2H), 1.37 (brs, 1H), 1.30 (s, 6H), 1.20 (s, 3H). ^13^C-NMR (CDCl_3_) δ 144.7, 141.4, 141.1, 140.2, 126.3, 126.2, 125.4, 125.4, 122.5, 112.8, 70.7, 43.7, 42.9, 29.9, 23.2. ESI-MS: 347.2 [M+Na], HRMS(ESI): Calcd. for C_19_H_24_F_3_O [M+H]: 325.1774, found: 325.1774.

*(S,3E,7E)-8-(Benzo[d]*[1,3]*dioxol-5-yl)-2,6-dimethyl-6-vinylocta-3,7-dien-2-ol* (**14g**) Yield: 38%, *R*_f_ = 0.26 (petroleum ether/ethyl acetate = 5:1), 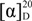 −10.2 (c 0.50, CHCl_3_). ^1^H-NMR (CDCl_3_) δ 6.91 (s, 1H), 6.77 (d, *J* = 8.0 Hz, 1H), 6.73 (d, *J* = 8.0 Hz, 1H), 6.22 (d, *J* = 16.2 Hz, 1H), 6.03 (d, *J* = 16.2 Hz, 1H), 5.93 (d, *J* = 1.0 Hz, 2H), 5.87 (dd, *J* = 17.5, 10.7 Hz, 1H), 5.68–5.52 (m, 2H), 5.05 (d, *J* = 10.7 Hz, 1H), 5.00 (d, *J* = 17.5 Hz, 1H), 2.21 (d, *J* = 6.5 Hz, 2H), 1.42 (brs, 1H), 1.29 (s, 6H), 1.16 (s, 3H). ^13^C-NMR (CDCl_3_) δ 147.9, 146.7, 145.3, 141.1, 135.7, 132.2, 127.0, 122. 9, 120.5, 112.3, 108.2, 105.4, 100.9, 70.7, 43.8, 42.6, 29.9, 29.7, 23.4. ESI-MS: 318.4 [M+NH_4_], HRMS (ESI): Calcd. for C_19_H_24_O_3_Na [M+Na]: 323.1618, found: 323.1639.

### 3.3. Activity Tests

The MTT assay was used to measure cell viability. All the tested compounds were dissolved in 0.5% DMSO. Before analysis, UMR 106 cells (rat osteosarcoma cell line) were cultured with or without different concentrations of compounds for 48 h, respectively. At the end of incubation 0.5% MTT (20 μL) was added to each well, followed by incubation at 37 °C in 5% CO_2_ atmosphere for 4 h. The medium was then removed carefully, and DMSO (150 μL) was added to each well. The plates were shaken gently for 10 min to dissolve the blue formazan crystals. Absorbance was measured at 570 nm using an ELx-800 universal microplate reader (Bio-Tek, Winooski, VT, USA).

## 4. Conclusions

In summary, a series of the analogues of ∆^3^-2-hydroxybakuchiol have been synthesized, and some of them have been found to possess evident cytotoxic activity against rat UMR106 cells. Furthermore, the SAR study gives a preliminary profile, including: (1) the chain tail on the natural product could be somewhat modified to enhance the activity; (2) the aromatic moiety or the terminal olefin attached on the main chain are allowed to be modified without evident loss of activity; (3) the stereoconfiguration at the quaternary chiral center has an important influence on the activity.
